# Palmitate inhibits arthritis by inducing *t-bet* and *gata-3* mRNA degradation in *i*NKT cells via IRE1α-dependent decay

**DOI:** 10.1038/s41598-017-14780-4

**Published:** 2017-11-02

**Authors:** Jae Sung Ko, Jae Moon Koh, Jae-Seon So, Yoon Kyung Jeon, Hye Young Kim, Doo Hyun Chung

**Affiliations:** 1 Department of Pathology, Seoul National University College of Medicine, Seoul, Korea; 2 Laboratory of Immune Regulation in Department of Biomedical Sciences, Seoul National University College of Medicine, Seoul, Korea; 3 Department of Medical Biotechnology, Dongguk University-Gyeongju, Gyeongju, Korea; 4 Laboratory of Mucosal Immunology in Department of Biomedical Sciences, Seoul National University College of Medicine, Seoul, Korea

## Abstract

Long chain fatty acids (LCFAs) exert pro-inflammatory effects *in vivo*. However, little is known regarding the effect of LCFAs on invariant (*i*) NKT cell functions. Here, we report an inhibitory effect of saturated LCFAs on transcription factors in *i*NKT cells. Among the saturated LCFAs, palmitic acid (PA) specifically inhibited IL-4 and IFN-γ production and reduced *gata-3* and *t-bet* transcript levels in *i*NKT cells during TCR-mediated activation. In *i*NKT cells, PA was localized and induced dilation in the endoplasmic reticulum and increased the mRNA levels of downstream molecules of IRE1α RNase. Moreover, PA increased the degradation rates of *gata-3* and *t-bet* mRNA, which was restored by IRE1α inhibition or transfection with mutant *gata-3* or *t-bet*, indicating that *gata-3* and *t-bet* are cleaved via regulated IRE1α-dependent decay (RIDD). A PA-rich diet and PA injection suppressed IL-4 and IFN-γ production by *i*NKT cells in C57BL/6, but not Jα18 knockout mice, which was restored by injection of STF083010, an IRE1α-specific inhibitor. Furthermore, a PA-rich diet and PA injection attenuated arthritis in an *i*NKT cell-dependent manner. Taken together, our experiments demonstrate that a saturated LCFA induced RIDD-mediated* t-bet* and *gata-3* mRNA degradation in *i*NKT cells, thereby suppressing arthritis.

## Introduction

Long chain fatty acids (LCFAs) are an abundant component of the Western diet and are proposed to induce various diseases including metabolic syndrome, cardiovascular disease, and inflammatory diseases. LCFAs are fatty acids that have aliphatic tails of 13 to 21 carbons and are classified as saturated and unsaturated, depending on their carbon- carbon single bond and double bond content. It has been shown that the structures, metabolism, and biological effects of LCFAs *in vivo* are very unique, and the absorption efficiency of dietary LCFAs depends on carbon chain length^1^. Moreover, restriction of saturated LCFA intake reduces the risk of cardiovascular diseases, as they promote inflammation in various organs^1^. In contrast, the American Heart Association recommends intake of unsaturated LCFA-rich foods for prevention of cardiovascular diseases based on the anti-inflammatory effects of LCFAs^2^. Thus, a biological balance between saturated and unsaturated LCFAs might be important for regulating various physiological and pathological events *in vivo*.

Several studies have reported that LCFAs affect various immune cell functions. O’Sullivan *et al*. demonstrated that de novo fatty acid synthesis was indispensable for the Th17 and regulatory T cell differentiation of CD4^+^ T cells, as well as the survival of CD8^+^ T cells^3,4^, indicating that intracellular fatty acid synthesis modulates certain functions of conventional CD4^+^ and CD8^+^ T cells. Moreover, among the exogenous LCFAs, palmitic acid upregulated the secretion of IL-1β and IL-18 via NLRP3 inflammasome activation in macrophages^5^ and activated dendritic cells to secret IL-1β in a toll-like receptor (TLR)4-dependent manner, thereby amplifying Th1/Th17 immune responses and inflammation^6,7^. These findings indicate that both intracellular fatty acids and extracellular LCFAs affect the functions of immune cells *in vitro* and *in vivo*.

Invariant natural killer T (*i*NKT) cells are a subset of T cells characterized by their recognition of the CD1d glycolipid antigen and their expression of the semi-invariant Vα14-Jα18 TCR^8^. α-galactosyl ceramide (α-GalCer), a glycolipid derived from a marine sponge activates *i*NKT cells *in vitro* and *in vivo* ^9^. Upon stimulation via TCR engagement, *i*NKT cells rapidly secrete large amounts of various cytokines, thereby playing an important role in the regulation of inflammatory diseases^10,11^. However, it has not been reported whether de novo fatty acids or extracellular saturated and unsaturated LCFAs affect the function of innate T cells including *i*NKT cells. Thus, we investigated effects of extracellular saturated and unsaturated LCFAs on *i*NKT cell function *in vitro* and *in vivo*. Our experiments demonstrate that saturated LCFAs inhibit IL-4 and IFN-γ production in *i*NKT cells by promoting *t-bet* and *gata-3* mRNA degradation via regulated IRE1α-dependent decay (RIDD), thereby attenuating *i*NKT cell-mediated joint inflammation.

## Results

### Palmitic acid inhibits IL-4 and IFN-γ, but not IL-2, IL-10, IL-13, or IL-17, production by *i*NKT cells *in vitro*

**Figure 1:**
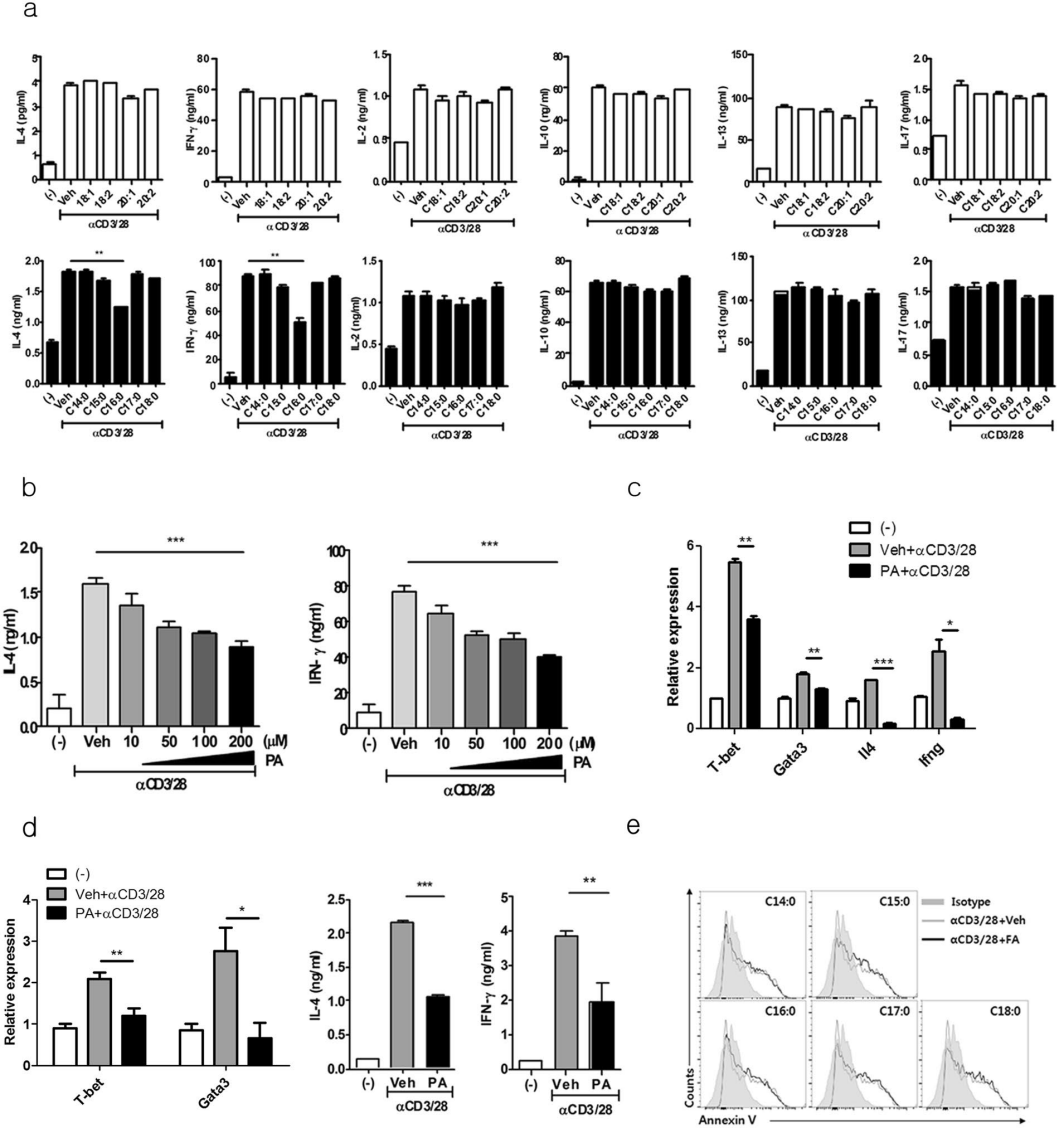
Palmitic acid (C16: 0), a saturated long chain fatty acid (LCFA), inhibits IL-4 and IFN-γ, but not IL-2, IL-10, IL-13, or IL-17 production by *i*NKT cells. (**a**) The levels of cytokines were measured in culture supernatants from an *i*NKT cell line treated with various saturated or unsaturated LCFAs in the presence of anti-CD3 and anti-CD28 monoclonal antibodies (mAbs) for 24 h. (**b**) The levels of IL-4 and IFN- γ in supernatant of *i*NKT cell line treated with various concentrations of palmitic acid (C16:0) in the presence of anti-CD3 and anti-CD28 mAb for 24 h. (**c**) The transcript levels of *t-bet*, *gata-3*, *Il4*, and *Ifng* were measured in the *i*NKT cell line. (**d**) α-GalCer/CD1d tetramer ^+^ TCRβ ^+^ *i*NKT cells were sorted from C57BL/6 mouse liver mononuclear cells and treated with palmitic acid and anti-CD3 and anti-CD28 mAbs for 24 h. The levels of *t-bet* and *gata-3* mRNA were measured in these cells, while IL-4 and IFN-γ levels were estimated in culture supernatants. (**e**) The expression levels of Annexin V in *i*NKT cells treated with various saturated LCFAs in the presence of anti-CD3 and anti-CD28 mAbs. *p < 0.05, **p < 0.01, ***p < 0.005.

To investigate whether saturated and unsaturated LCFAs regulate the function of *i*NKT cells, we treated mouse *i*NKT cell line with various saturated and unsaturated LCFAs in the presence of anti-CD3 and CD28 monoclonal antibodies (mAbs) or α-GalCer-loaded bone marrow-derived dendritic cells (BMDCs). Among the saturated LCFAs, only palmitic acid (C16:0) inhibited IL-4 and IFN-γ production by *i*NKT cell line in a dose-dependent manner, whereas it did not alter production of IL-2, IL-10, IL-13, and IL-17 (Fig. 1a and b, Supplementary Fig. 1). Kinetic analysis revealed that palmitic acid gradually reduced IL-4 and IFN-γ, but not IL-2, IL-10, IL-13, and IL-17 levels in the culture supernatant (Supplementary Fig. 2a). In contrast, no unsaturated LCFAs affect the production of any of the cytokines evaluated in the *i*NKT cell line (Fig. 1a). Palmitic acid reduced the mRNA levels of *Ifng* and *Il4* as well as those of their signature transcription factors such including *t-bet* and *gata-3* upon TCR stimulation (Fig. 1c). Consistent with these *i*NKT cell line experiments, the palmitic acid-induced inhibition of IL-4 and IFN-γ production and reductions in *t-bet* and *gata-3* mRNA expression levels were also found in CD1d/α-GalCer^+^ *i*NKT cells sorted from liver mononuclear cells of C57BL/6 mice (Fig. 1d). However, palmitic acid did not affect apoptosis in the *i*NKT cell line in the presence of TCR stimulation (Fig. 1e). These findings suggest that palmitic acid, a saturated LCFA, inhibits IL-4 and IFN-γ production by suppressing *t-bet* and *gata-3* mRNA levels in *i*NKT cells upon TCR stimulation.

### Palmitic acid is localized in the endoplasmic reticulum (ER) and induces ER stress in *i*NKT cells

**Figure 2:**
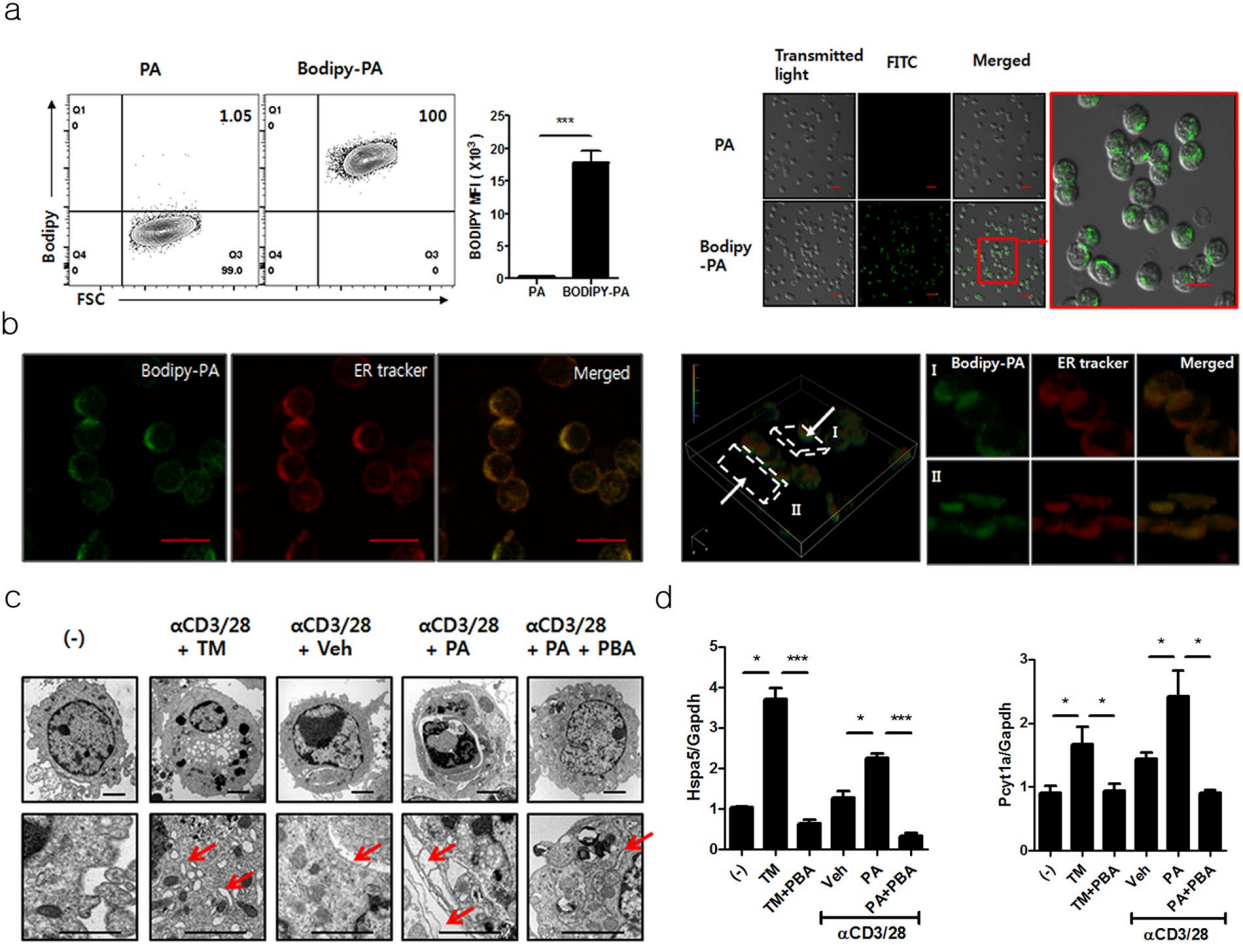
Palmitic acid was localized in the endoplasmic reticulum (ER) in *i*NKT cells, and it induced ER stress. (**a**) *i*NKT cells were treated with Bodipy-conjugated or unconjugated palmitic acid for 2 min and were analyzed using flow cytometry or confocal microscopy. The bars indicate 20 μm and 10 μm in low- and high-power images, respectively. (**b**) Z-stacks were used for three-dimensional reconstruction of *i*NKT cells treated with Bodipy-conjugated palmitic acid and an ER tracker under confocal microscopic examination. The bars indicate 10 μm. (**c** and **d**) Upon stimulation with anti-CD3 and CD28 mAbs, (**c**) electron microscopic examination was performed, and (**d**) the levels of *Hspa5* and *Pcyt1* mRNA were measured in the *i*NKT cells treated with tunicamycin or palmitic acid in the presence or absence of 4-phenylbutyrate (PBA). The bars and arrows indicate 2 μm and ER, respectively. *p < 0.05, ***p < 0.005.

To investigate the mechanisms by which palmitic acid inhibited IL-4 and IFN-γ production in *i*NKT cells, the *i*NKT cell line was treated with Bodipy-conjugated palmitic acid. Flow cytometry and confocal microscopic examination revealed that palmitic acid was located in the cytoplasm rather than in membrane or nucleus (Fig. 2a). Furthermore, on confocal microscopic examination, palmitic acid was localized in the ER compartment (Fig. 2b), suggesting that it may regulate cellular function by modifying ER homeostasis. Consistently, electron microscopic examination revealed palmitic acid-induced dilation and elongation of the ER (Fig. 2c), and we also found increased transcription levels of ER chaperone BiP (*Hspa5*) and enzyme involved in ER dilation CCTα (*Pcyt1a*) in *i*NKT cells upon TCR stimulation (Fig. 2d). These results were similar to those obtained from *i*NKT cells treated with tunicamycin, a potent ER stress inducer (Fig. 2c and d). Moreover, an ER stress inhibitor, 4-phenylbutyrate (PBA) restored palmitic acid-induced dilation of the ER and reduced the transcription levels of *Hspa5* and *Pcyt1a* in *i*NKT cells (Fig. 2d). These results indicate that influx of palmitic acid into the cytoplasm induces ER modulation in *i*NKT cells during TCR stimulation.

### Palmitic acid-mediated ER stress suppresses IL-4 and IFN-γ in *i*NKT cells via the IRE1α pathway

**Figure 3:**
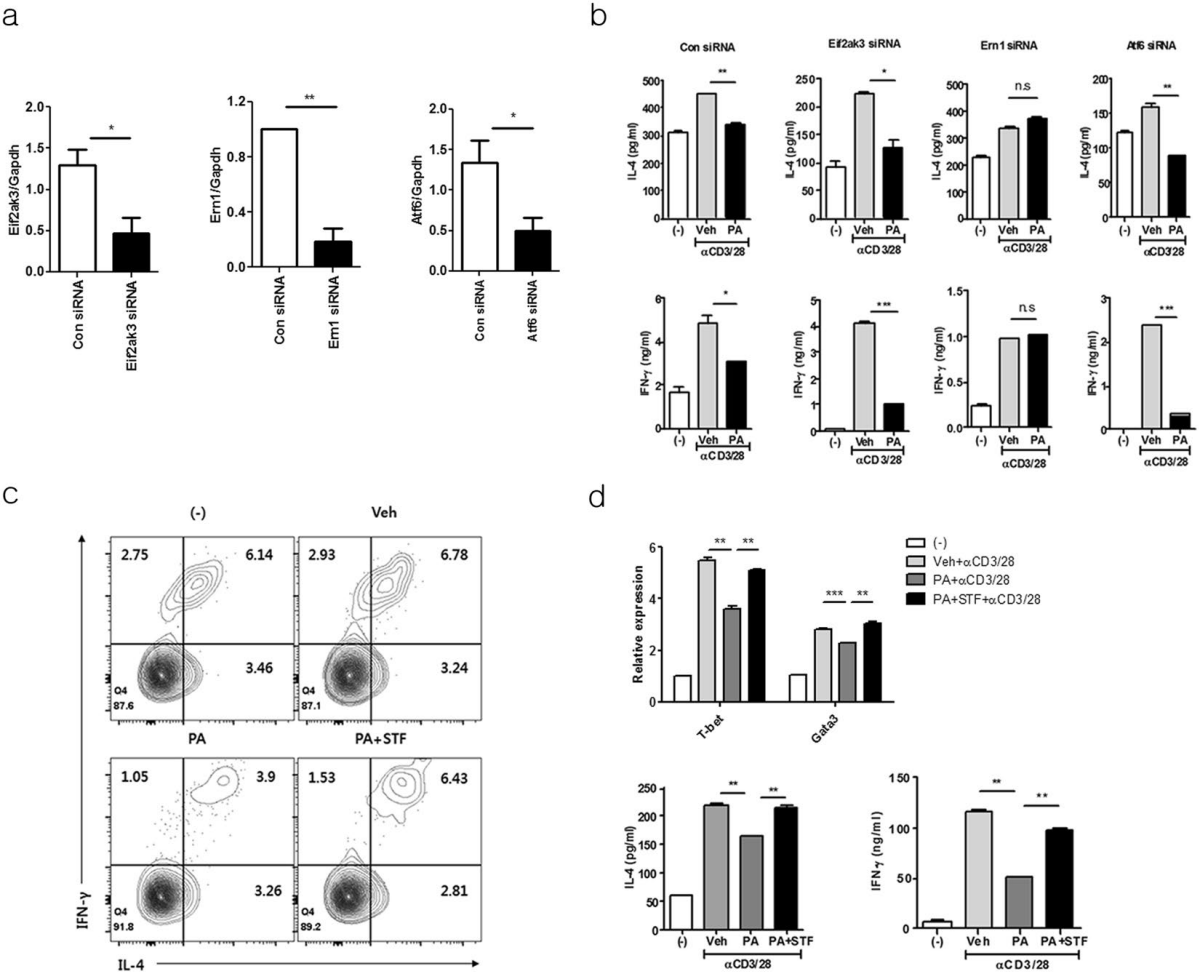
Palmitic acid-mediated ER stress suppresses IL-4 and IFN-γ production by *i*NKT cells via the IRE1α pathway. (**a** and **b**) *i*NKT cells were transfected with siRNA for control, *Eif2ak3*, *Ern1*, or *Atf6* and treated with palmitic acid or vehicle in the presence of anti-CD3 and anti-CD28 mAbs for 24 h. (**a**) The efficiency of knockdown was estimated for each individual gene. (**b**) The levels of IL-4 and IFN-γ were measured in culture supernatants using ELISA. (c and d) *i*NKT cells were incubated with palmitic acid or vehicle in the presence of anti-CD3 and anti-CD28 mAbs for 24 h. To inhibit IRE1α, STF083010 was added during palmitic acid treatment. (**c**) The levels of cytosolic IL-4 and IFN- γ were estimated using cytometric analysis. (**d**) The transcription levels of *t-bet* and *gata-3* were measured in the *i*NKT cell line using real time PCR, while the levels of IL-4 and IFN- γ were quantified using ELISA. *p < 0.05, **p < 0.01, ***p < 0.005.

**Figure 4:**
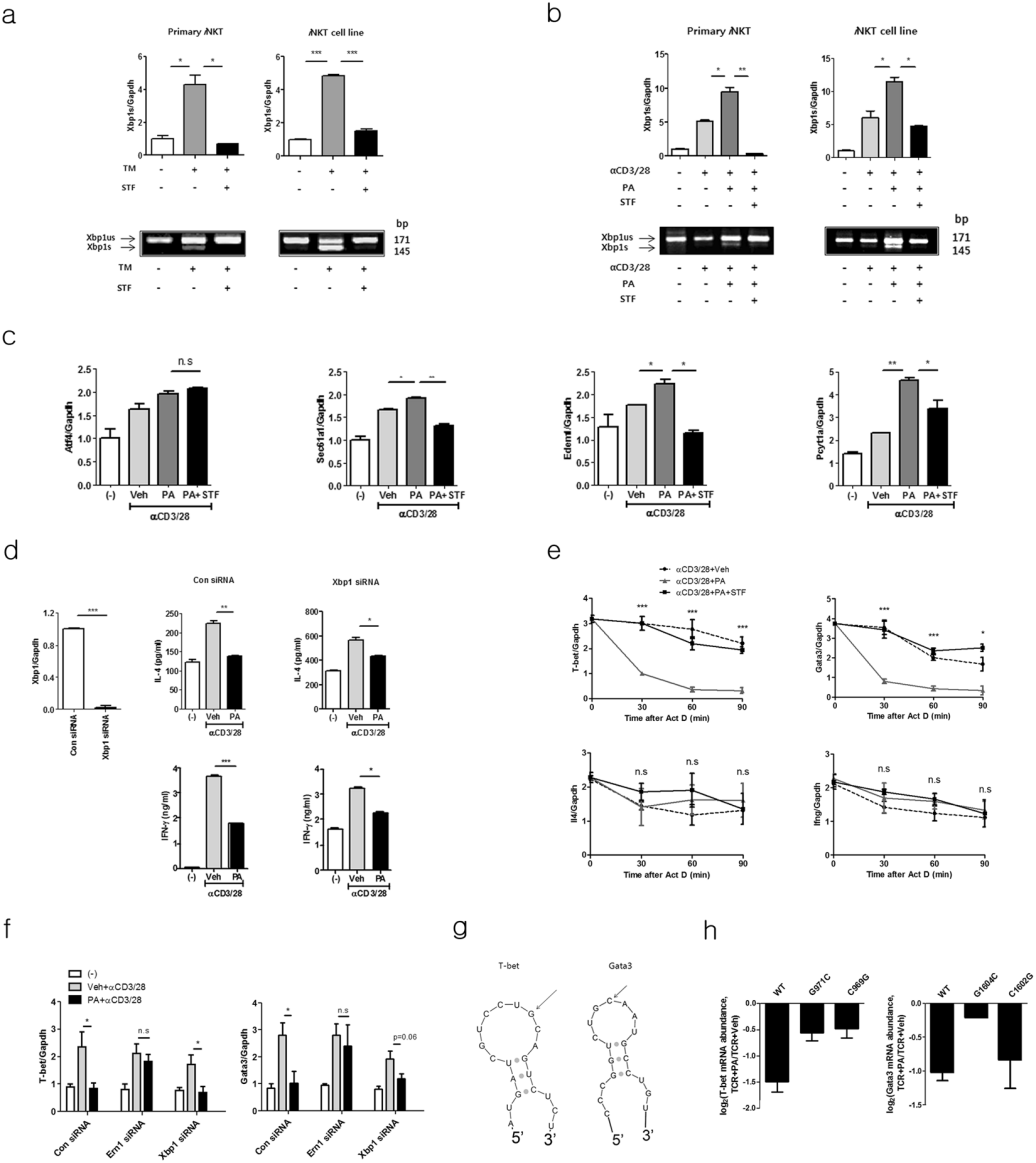
Palmitic acid promotes the degradation of *t-bet* and *gata-3* mRNA in *i*NKT cells via regulated IRE1α-dependent decay (RIDD), thereby suppressing IL-4 and IFN- γ production. (**a** and **b**) *i*NKT cells or α-GalCer/CD1d tetramer ^+^ TCRβ ^+^ *i*NKT cells sorted from C57BL/6 mouse liver mononuclear cells were treated with (**a**) tunicamycin (TM) and/or STF083010 or (**b**) palmitic acid and/or STF083010 in the presence of anti-CD3 and anti-CD28 mAbs. Reverse-transcription PCR and real-time PCR were performed to estimate un-spliced (us) or spliced (s) *xbp-1*. (**c**) The expression levels of *Sec* *61a1*, *Edem1*, *Pcyt1a* and *Atf4* were also measured in *i*NKT cells using real-time PCR. (**d**) To knock-down *xbp-1*, *i*NKT cells were transfected with control or *xbp-1* siRNA and then treated with palmitic acid or vehicle in the presence of anti-CD3 and anti-CD28 mAbs for 24 h. The levels of IL-4 and IFN- γ were estimated in culture supernatants using ELISA. (**e**) The transcription levels of *t-bet*, *gata-3*, *Il4*, and *Ifng* were estimated in *i*NKT cells treated with vehicle, palmitic acid, or palmitic acid and STF083010 in the presence of actinomycin D at the indicated time points. (**f**) The transcription levels of *t-bet*and *gata-3* were measured in *Ern1*- or *xbp-1*-knockdown or control *i*NKT cells upon treatment with vehicle, palmitic acid, or palmitic acid and STF083010. (**g**) mRNA structural modeling for* t-bet* and *gata-3*. (**h**) DN32.D3 cells were transfected with mutant *t-bet* (G971C or C969G), mutant *gata-3* (G1604C or C1602G), or wild type *t-bet* and *gata-3* and then treated with palmitic acid or vehicle in the presence of anti-CD3 and anti-CD28 mAbs. mRNA measurement reflects the amount of relative degradation of *t-bet* or *gata-3* transcript. n.s. not significant, *p < 0.05, **p < 0.01, ***p < 0.005.

During ER stress, three transmembrane proteins, protein kinase R-like ER-localized eIF2α kinase (PERK), inositol-requiring enzyme-1 (IRE-1), and activating transcription factor 6 (ATF6), are typically activated in mammalian cells^12^. Thus, to explore the activation pathways involved in palmitic acid-mediated inhibition of IL-4 and IFN-γ production in *i*NKT cells, we knocked down these molecules by siRNA transfection in the *i*NKT cell line (Fig. 3a). Among the three ER stress activation molecules, knockdown of IRE1α (*Ern1*) restored IL-4 and IFN-γ production during TCR stimulation, whereas down-regulation of PERK (*Eif2ak3*) and ATF6 (*Atf6*) did not affect the levels of these cytokines compared with controls (Fig. 3b). These findings indicate that the IRE1α pathway is involved in palmitic acid-induced inhibition of IL-4 and IFN-γ production by *i*NKT cells. Consistent with this, STF083010, an IRE1α-specific inhibitor, restored the production of IL-4 and IFN-γ as well as mRNA levels of *t-bet* and *gata-3* in *i*NKT cells treated with palmitic acid in the presence of TCR stimulation (Fig. 3c and d). It has been reported that ER stress-induced activation of IRE1α exerts endonuclease activity on various mRNAs, including *xbp-1*, thereby generating the spliced form of XBP1(Bettigole and Glimcher, 2015). Thus, we measured the mRNA levels of spliced *xbp-1* in *i*NKT cells treated with palmitic acid and/or anti-CD3 and anti-CD28 mAbs. Similar to tunicamycin treatment, palmitic acid treatment in the presence of TCR stimulation increased the transcription of spliced *xbp-1* in the *i*NKT cell line and in sorted *i*NKT cells (Fig. 4a and b). Moreover, STF083010 reduced transcription levels of spliced *xbp-1* in palmitic acid and tunicmycin-treated *i*NKT cells (Fig. 4a and b). Consistent with these results, in *i*NKT cells, palmitic acid in the presence of TCR stimulation gradually increased the expression levels of the molecules downstream of spliced XBP1, including *Sec* *61a1*, *Edem1*, and *Pcyt1a*, which were also decreased by STF083010 (Fig. 4c and Supplementary Fig. 2b). In contrast, mRNA levels of ATF4-related genes were increased, peaked at 8 h, and abruptly decreased, which was not consistent with cytokine production (Supplementary Fig. 2b). Altogether, these results indicate that palmitic acid inhibits IL-4 and IFN-γ production by activating the IRE1α pathway in the ER of *i*NKT cells.

### Palmitic acid induces degradation of *t-bet* and *gata-3* mRNA via RIDD, thereby suppressing IL-4 and IFN-γ production in *i*NKT cells

Our experiments demonstrated that palmitic acid activated both IRE1α and XBP1 in *i*NKT cells in the presence of TCR stimulation. Thus, to determine which of the protein directly regulates the palmitic acid-induced inhibition of IL-4 and IFN-γ production by *i*NKT cells, we knocked downed IRE1α and XBP1 in the *i*NKT cell line using siRNA. In contrast to IRE1α (Fig. 3b), knockdown of *xbp-1* did not alter palmitic acid-induced inhibition of IL-4 and IFN-γ production in *i*NKT cells treated with anti-CD3 and CD28 mAbs (Fig. 4d). These results indicate that activation of IRE1α rather than XBP1 plays a critical role in palmitic acid-induced inhibition of IL-4 and IFN-γ production by *i*NKT cells. Furthermore, IRE1α exerts RNase activity to regulate cellular functions by degrading various mRNA molecules via RIDD during ER stress^13,14^. To explore whether palmitic acid suppresses IL-4 and IFN-γ production by *i*NKT cells via RIDD, *i*NKT cells were treated with actinomycin D in the presence of anti-CD3 and anti-CD28 mAbs. Interestingly, palmitic acid significantly decreased the transcript levels of* t-bet* and *gata-3*, but not *Ifng* and *Il4* in *i*NKT cells compared with the control group, and these levels were restored by STF083010 treatment (Fig. 4e). Consistent with these results, knockdown of *Ern* *1* restored *t-bet* and *gata-3* transcript levels in *i*NKT cells treated with palmitic acid and anti-CD3 and anti-CD28 mAbs compared with control siRNA-treated *i*NKT cells; however, *xbp-1* knockdown did not affect reduction of two molecules (Fig. 4f). Generally, the target cleavage sites of the endonuclease IRE1α are located in the small stem loop of hairpin structures^13,14^. Structural mRNA modeling demonstrated that both *t-bet* and *gata-3* contain an IRE1α-cleavage site in the loop of a hairpin structure (Fig. 4g). To confirm this, we transfected DN32.D3 cells, a NKT cell hybridoma, with wild type (WT) or mutated* t-bet* and *gata-3* as described^15^. Palmitic acid suppressed *t-bet* and* gata-3* transcript levels in DN32.D3 cells transfected with WT *t-bet* or *gata-3* compared with vehicles. In contrast, palmitic acid minimally inhibit transcript levels of *t-bet* or *gata-3* in DN32D.3 cells transfected with two types of *t-bet* mutant (G971C or C969G) or a *gata-3* mutant (G1604C), but did those of *gata-3* in cells transfected with C1602G-mutated *gata-3*. These findings suggest that *t-bet* and *gata-3* are palmitic acid-mediated RIDD substrate in *i*NKT cells (Fig. 4h). Combined, these findings indicate that palmitic acid inhibits IL-4 and IFN-γ production by degrading *t-bet* and *gata-3* mRNA via RIDD in *i*NKT cells.

### Palmitic acid inhibits IL-4 and IFN-γ production by* i*NKT cells upon TCR stimulation via ER stress *in vivo*

**Figure 5:**
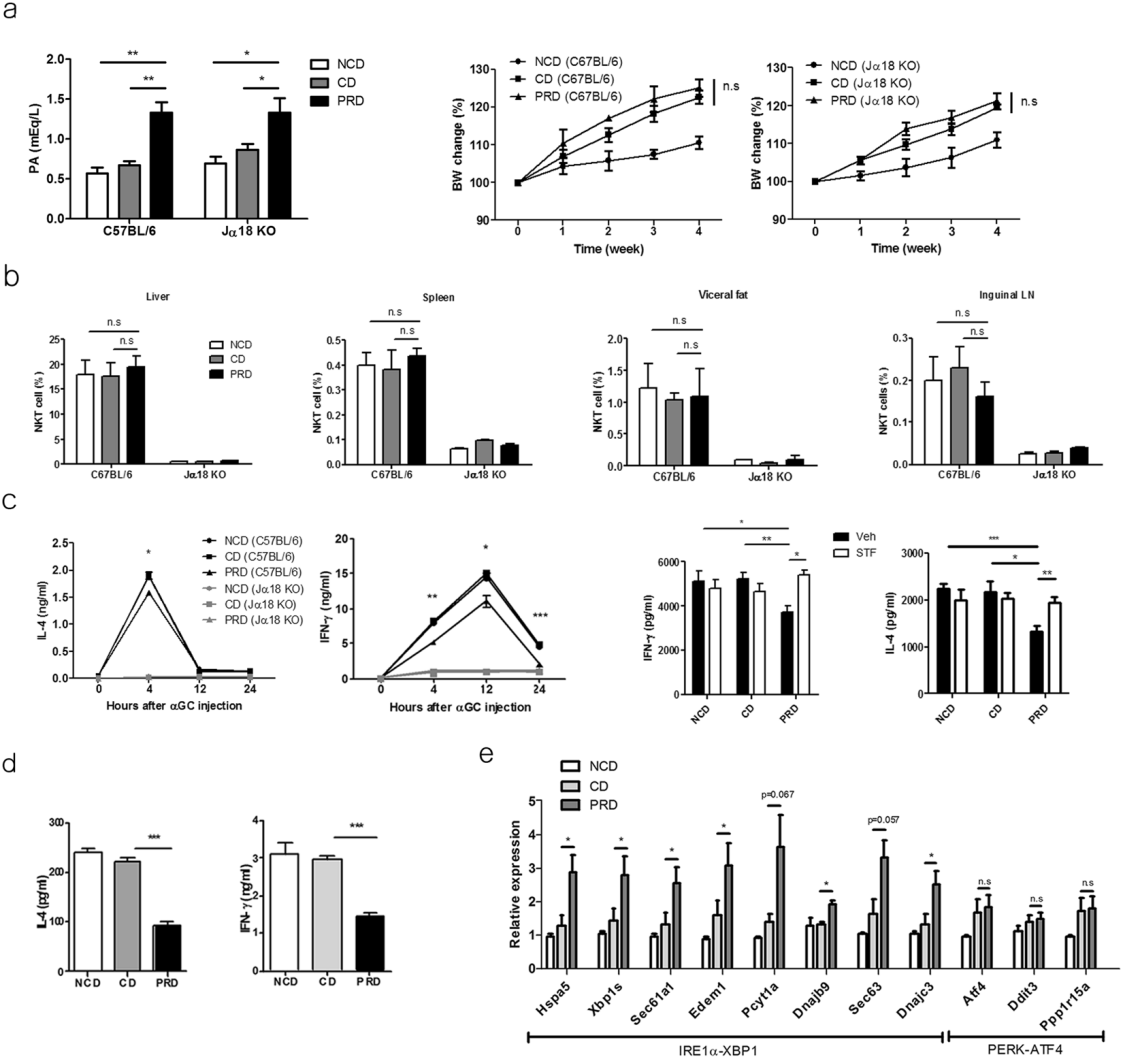
Dietary palmitic acid inhibits IL-4 and IFN- γ production by *i*NKT cells via ER stress in the presence of TCR stimulation *in vivo*. (**a**) Serum levels of palmitic acid were estimated in C57BL/6 mice fed a normal chow diet (NCD), palmitic acid-rich diet (PRD), or a control diet (CD) for 4 weeks. The CD contained similar amount of calories as those of the PRD, but minimal palmitic acid. The serum concentration of palmitic acid and body weight of C57BL/6 and Jα18 Knockout (KO) mice were measured. (**b**) The percentages of *i*NKT cells in the visceral fat, liver, spleen, and inguinal lymph nodes were analyzed. The levels of IL-4 and IFN-γ in (**c**) the serum of C57BL/6 or Jα18 KO mice fed the NCD, CD, or PRD in the presence of (right panel) or absence (left panel) intraperitoneal STF083010 (20 mg/kg) injection every week for 4 weeks after α-GalCer injection and in (**d**) the supernatant of hepatic mononuclear cells from these mice in the presence of α-GalCer. (**e**) The expression levels of *Hspa5*, spliced *xbp-1*, *Sec* *61a1*, *Edem1*,* Pcyt1a*, *Dnajb9*, *Sec* *63*, *Dnajc3*, *Atf4*, *Ddit3*, and *Ppp1r15a* in hepatic *i*NKT cells obtained from C57BL/6 mice fed the NCD, CD, or PRD for 1 week using real time PCR. n = 12 per group in a; n = 6 per group in b, d and e; n = 9 per group in c. Data were pooled from three independent experiments and analyzed. *p < 0.05, **p < 0.01, ***p < 0.005.

To explore whether palmitic acid inhibits IL-4 and IFN-γ production by *i*NKT cells *in vivo*, we injected C57BL/6 mice with bovine serum albumin (BSA)-conjugated palmitic acid. The serum levels of palmitic acid peaked at 4 h, after which they decreased gradually (Supplementary Fig. 3a), and the majority of hepatic *i*NKT cells contained Bodipy-conjugated palmitic acid in the cytosol (Supplementary Fig. 3b). The expression levels of *Hspa5*, spliced *xbp-1*, *Sec* *61a1*, *Edem1*, *Pcyt1a*, *Dnajb9*, *Sec* *63*, and *Dnajc3* were increased in *i*NKT cells obtained from tunicamycin or palmitic acid-injected C57BL/6 mice (Supplementary Fig. 3c and d). Upon STF083010 injection, the increased levels of these molecules were suppressed in *i*NKT cells obtained from palmitic acid-injected C57BL/6 mice. In contrast, the transcript levels of *Atf4*, *Ddit3*, and *Ppp1r15a* were not altered in hepatic *i*NKT cells from C57BL/6 mice injected BSA-conjugated palmitic acid (Supplementary Fig. 3d). Moreover, the serum levels of IL-4 and IFN-γ were lower in C57BL/6, but not in Jα18 knockout (KO), mice injected with BSA-conjugated palmitic acid compared with mice treated with vehicle 4 h after α-GalCer administration. Injection of STF083010 restored IL-4 and IFN-γ levels (Supplementary Fig. 3e and f). Altogether, these findings indicate that palmitic acid inhibits IL-4 and IFN-γ production by *i*NKT cells via ER stress in C57BL/6 mice. To confirm the effect of palmitic acid on *i*NKT cell functions under physiological conditions *in vivo*, C57BL/6 and Jα18 KO mice were fed a palmitic acid-rich diet (PRD) or a control diet (CD), which contained minimal levels of palmitic acid but a similar numbers of calories as those of the PRD. The body weight of the mice and the numbers and tissue distribution of *i*NKT cells were similar between the two treatment groups; however, the levels of palmitic acid were higher in the serum of C57BL/6 and Jα18 KO mice fed a PRD compared with those fed the CD or normal chow diet (NCD) (Fig. 5a and b). Injection of α-GalCer resulted in lower serum levels of IL-4 and IFN-γ in C57BL/6 mice fed the PRD compared with mice fed the CD, which was restored by STF083010 injection; however, these results were not found in Jα18 KO mice (Fig. 5c). Upon stimulation with α-GalCer, mononuclear cells from the livers of C57BL/6 mice fed the PRD produced less IL-4 and IFN-γ than did those from mice fed the CD (Fig. 5d). Moreover, the expression levels of *Hspa5*, spliced *xbp-1*, *Sec* *61a1*, *Edem1*, *Pcyt1a*, *Dnajc3*, *Sec* *63*, and *Dnajc3* were increased in *i*NKT cells obtained from C57BL/6 mice fed the PRD compared with mice fed the CD, whereas levels of *Atf4*, *Ddit3*, and *Ppp1r15a* transcript were not altered (Fig. 5e). These findings indicate that dietary palmitic acid induces ER stress in *i*NKT cells *in vivo*, thereby inhibiting IL-4 and IFN-γ production.

### Dietary palmitic acid attenuates antibody-induced joint inflammation by inhibiting IL-4 and IFN-γ production by *i*NKT cells

**Figure 6:**
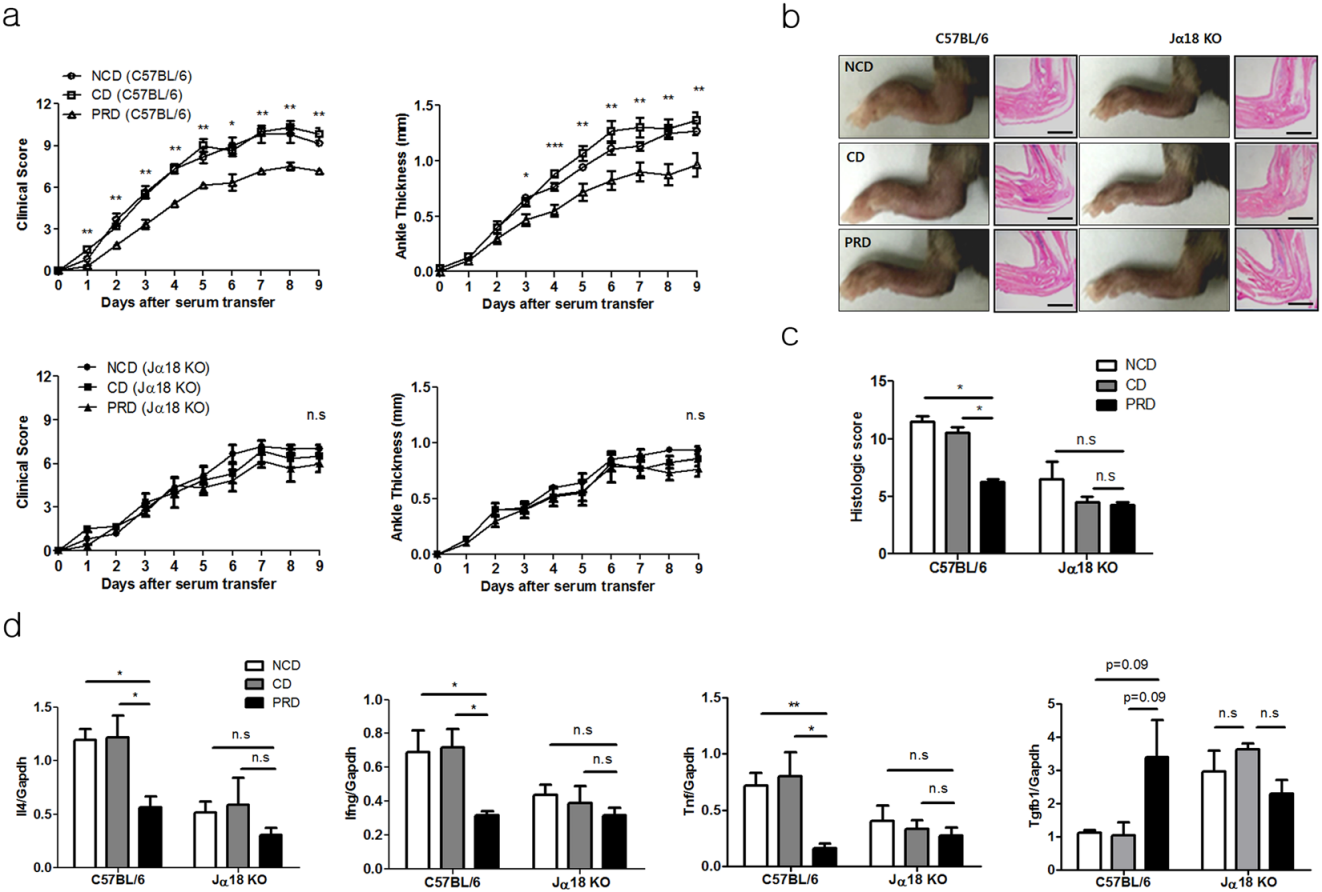
Dietary palmitic acid suppresses antibody-induced joint inflammation by inhibiting IL-4 and IFN-γ production. (**a**–**d**) C57BL/6 and Jα18 KO mice were fed a NCD, CD, or PRD for 6 weeks, and joint inflammation was induced by K/BxN serum injection. (**a**) The ankle thickness and clinical scores were measured during antibody-induced arthritis. (**b**) The gross and microscopic images of the ankles of these mice are presented. The bars indicate 2 mm. (**c**) The histological scores of joint inflammation were measured. (**d**) The expression levels of *Il4*,* Ifng*, *Tnf*, and *Tgfb1* were measured in the joints of these mice during antibody-induced arthritis. n = 10 per group in **a–d**. Data were pooled from two independent experiments and analyzed. *p < 0.05, **p < 0.01, ***p < 0.005.

**Figure 7:**
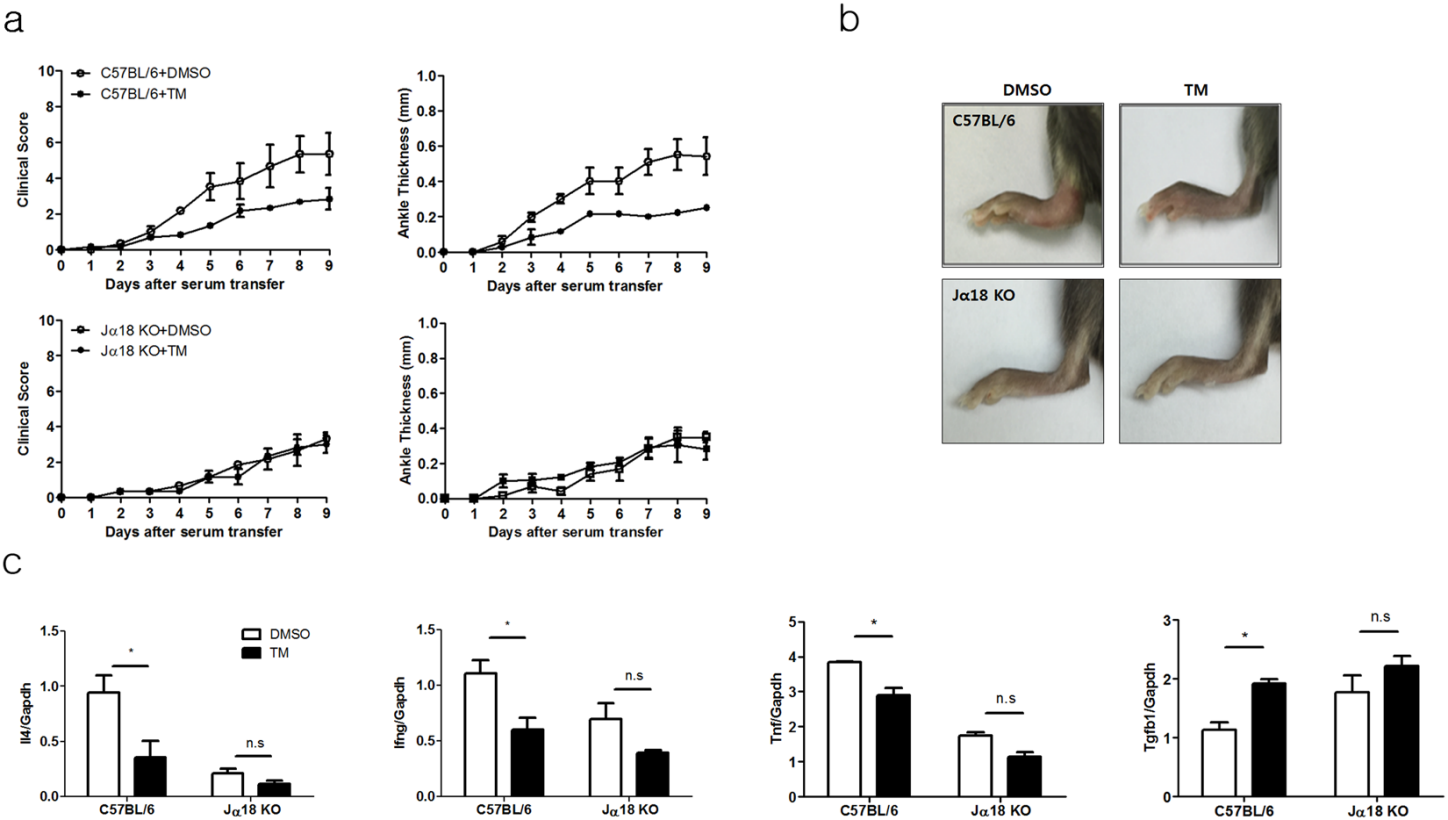
An ER stress inducer tunicamycin, suppresses antibody-induced joint inflammation by inhibiting IL-4 and IFN-γ production. (**a**–**c**) C57BL/6 and Jα18 KO mice were injected with tunicamycin (0.3 mg/kg) every 5 days (days 0 and 5), and joint inflammation was induced by K/BxN serum injection. (**a**) The ankle thickness and clinical scores were measured in C57BL/6 and Jα18 KO mice during antibody-induced arthritis. (**b**) The gross images of the ankles of these mice are presented. (**c**) The expression levels of *Il4*,* Ifng*, *Tnf*, and*Tgfb1* were measured in the joints of these mice during antibody-induced arthritis. n = 10 per group in **a–c**. Data were pooled from two independent experiments and analyzed. *p < 0.05, **p < 0.01, ***p < 0.005.

To confirm the inhibitory effect of palmitic acid on IL-4 and IFN-γ production by *i*NKT cells in a disease model, we induced joint inflammation in C57BL/6 and Jα18 KO mice via administration of K/BxN serum and subsequently treated these mice with palmitic acid or vehicle. Compared with controls, injection with BSA-conjugated palmitic acid attenuated joint inflammation in C57BL/6 mice but not in Jα18 KO mice (Supplementary Fig. 4a–c). Moreover, adoptive transfer of *i*NKT cells into Jα18 KO mice restored joint inflammation, which was inhibited by injecting BSA-conjugated palmitic acid, but not vehicle. Injection of palmitic acid, compared with vehicle, decreased the levels of IL-4, IFN-γ, and TNF-α in joint tissues in C57BL/6 and Jα18 KO mice with adoptive *i*NKT cell transfer, whereas it increased TGF-β levels in the joints of these mice (Supplementary Fig. 4d). Consistently, PRD-fed C57BL/6 mice showed less joint inflammation compared with mice fed a CD or NCD. In contrast, attenuation of joint inflammation was not detected in Jα18 KO mice fed the PRD compared with those fed the CD or NCD (Fig. 6a–c). Higher levels of IL-4, IFN-γ, and TNF-α, but lower levels of TGF-β, were detected in joint tissues of C57BL/6 mice fed the CD or NCD compared with those fed the PRD. However, the PRD did not alter cytokine levels in the joints of Jα18 KO mice (Fig. 6d). Consistent with the effect of palmitic acid on arthritis, tunicamycin also suppressed joint inflammation in C57BL/6 mice, but not in Jα18 KO mice (Fig. 7). Moreover, joint inflammation was similarly developed in C57BL/6 mice fed NCD, CD, or PRD for 4 weeks, and then continuously NCD for 2 weeks, suggesting that suppressive effect of palmitic acid on *i*NKT cell function might be reversible (Supplementary Fig. 5a–d). Taken together, these results indicate that dietary palmitic acid suppresses joint inflammation by inhibiting IL-4 and IFN-γ production by *i*NKT cells.

## Discussion

A growing body of evidence indicates the inflammatory effects of the saturated LCFA palmitic acid on various cell types and in many diseases. Several studies have demonstrated that palmitic acid promotes inflammatory processes in islet β cells and macrophages via the TLR4/MyD88 pathway and NLRP3-ASC inflammasome activation, thereby affecting insulin sensitivity^5,16^. This palmitic acid-induced inflammatory response was synergistically induced with lipopolysaccharide via *de novo* ceramide biosynthesis in macrophages^17^. Moreover, palmitic acid also acts as a pro-inflammatory factor in various diseases including arthritis, atherosclerosis, and hypothalamic dysregulation^18–20^. In particular, palmitic acid upregulated IL-6 in human chondrocytes and fibroblast-like synovial cells via TLR4 signaling in an arthritis model^18^. In contrast to this pro-inflammatory effect, our experiments clearly demonstrated that palmitic acid attenuated antibody-induced arthritis by inducing ER stress in *i*NKT cells. Thus, opposing effects of palmitic acid on inflammation suggest that palmitic acid exerts different effects depending on the microenvironment of the target and on the cell types and signaling pathways involved.

Among the immune cells, T cells increase the ER stress-associated unfolded protein response (UPR) and expression levels of GRP78 in a Ca^2+^-dependent manner during TCR-mediated activation^21,22^. Furthermore, GRP78 deficiency reduces the proliferation of granzyme B-mediated cytotoxicity of CD8^+^ T cells^23^. These findings indicate that ER stress modulates the function and viability of conventional CD4^+^ and CD8^+^ T cells. However, it is not known whether the metabolic microenvironment affects the function of immune cells by modulating ER homeostasis, particularly in innate T-lineage *i*NKT cells. Our experiments demonstrated that extracellular palmitic acid is translocated into the cytosol and localized in the ER, and that it induces elongation of the ER and increased the expression levels of GRP78 (also known as BiP) in activated *i*NKT cells. Moreover, palmitic acid inhibited IL-4 and IFN-γ production by *i*NKT cells in the presence of TCR stimulation. Consistent with these results, the transcription levels of *gata-3* and *t-bet* in activated *i*NKT cells were reduced by palmitic acid treatment, suggesting that the expression of these transcription factors might be modulated by palmitic acid. Several studies have demonstrated that palmitic acid induces ER stress and promotes interaction of ATF4 and CREB1 with the atf4 promoter in non-immune cells via activation of the PERK pathway, thereby regulating cell death^24–26^. Moreover, palmitic acid attenuated leptin and insulin-like growth factor 1 expression in the brain by activating C/EBPa homologous protein (CHOP)^27^. These findings indicate that palmitic acid activates the PERK pathway, rather than IRE1α in non-immune cells during ER stress. In contrast to these results, palmitic acid affected the cytokine production by, but not the viability of, *i*NKT cells during TCR-mediated activation via IRE1α, but not the PERK pathway. IRE1α is the most conserved mediator of the UPR, which increases its C-terminal endoribonuclease activity via phosphorylation-induced conformation shifts, thereby inducing RIDD and generating spliced XBP1 (Bettigole and Glimcher, 2015). RIDD contributes to either preservation of ER homeostasis or induction of cell death by site specific degradation of ER-localized mRNAs (Maurel *et al*., 2014). In our experiments, palmitic acid enhanced IRE1α endonuclease-dependent degradation of *t-bet* and *gata-3* mRNA, but not of *Il4* and *Ifng* transcripts in *i*NKT cells. These results indicate that palmitic acid inhibits IL-4 and IFN-γ production by *i*NKT cells via RIDD-mediated degradation of *t-bet* and *gata-3* mRNA. Moreover, RIDD induces cleavage of mRNAs containing the XBP-1 consensus site in all species, and it is therefore considered as a sequence- and structure-specific cellular event^13,14^. In our study, structural modeling revealed that *t-bet* and *gata-3* mRNA contain the XBP-1 consensus site in their hairpin structure loops (Fig. 4g). Thus, we here demonstrated that a saturated LCFA promotes degradation of *t-bet* and *gata-3* mRNA in T-lineage cells via RIDD during TCR-mediated activation, resulting in suppression of IL-4 and IFN-γ production. These results suggest that extracellular metabolites can affect the transcriptional activity of target proteins in immune cells *in vivo* and highlight the implications of RIDD in the regulation of immune cells by inhibition of transcription factors. Furthermore, based on these results, we also hypothesize that palmitic acid might affect Th1 and/or Th2 cell differentiation *in vivo*, although further research is warranted to clarify this.

In our experiments, tunicamycin also suppressed IL-4 and IFN-γ production by *i*NKT cells. Moreover, it has been reported that tunicamycin may act through palmitoylation as well as induction of ER stress^28^, which led to consider palmitic acid-mediated palmitoylation effect on *i*NKT cells *in vitro* and *in vivo*. However, palmitic acid increased the transcript levels of spliced *xbp-1* and its downstream genes during TCR-mediated activation in *i*NKT cells, and this effect was reduced by treatment with an IRE1α-specific inhibitor. These results indicate that spliced XBP1 was activated in *i*NKT cells in the palmitic acid-induced ER response rather than palmitoylation. The IRE1α-mediated generation of spliced XBP1, a potent transcription factor, exerts critical effects on differentiation, lipid metabolism, pro-inflammatory cytokine production, and the HIF-1α-dependent hypoxia pathway in various cell types^12,29–31^. However, the functional role of spliced XBP1 has not been well characterized in immune cells. Recent studies have demonstrated that XBP1 deficiency induces defects in eosinophil differentiation and functions of CD8^+^ and tumor-associated dendritic cells^32–34^, indicating that spliced XBP1 plays a critical role in development and function of immune cells. However, spliced XBP1 might be play a minimal role in palmitic acid-induced inhibition of IL-4 and IFN-γ production by *i*NKT cells, although the functional role of spliced XBP1 in *i*NKT cells remains unknown. In recent years, it has been reported that altered IRE1α function is associated with various diseases including cancers, diabetes, and inflammatory and neurodegenerative disorders^35–37^. Interestingly, ER stress-related gene signatures were increased in synovial cells in rheumatoid arthritis and in deficiency of GRP78-inhibited collagen-induced arthritis^38^, and deletion of IRE1α in myeloid cells protected mice from arthritis in a K/BxN serum transfer model^37^. Consistent with our results, these findings suggest that the IRE1α-mediated UPR in immune cells might be involved in the regulation of joint inflammation.

In conclusion, our experiments demonstrate that the saturated LCFA palmitic acid inhibits IL-4 and IFN-γ production in *i*NKT cells by promoting *t-bet* and *gata-3* mRNA degradation via RIDD, thereby regulating *i*NKT cell-mediated diseases. This study highlights the effect of saturated LCFAs on *i*NKT cell-mediated immune regulation via ER stress *in vivo*.

## Materials And Methods

### Mice

C57BL/6 mice (7–8 weeks old) were purchased from Orient Company Ltd. (Seoul, Korea). Jα18^-/-^ mice were a gift from Dr. M. Taniguchi (Chiba University, Chiba, Japan). KRN TCR transgenic mice and NOD mice, gifts from Drs. D. Mathis and C. Benoist (Harvard Medical School, Boston, MA, USA and the Institut de Genetique et de Biologie Moleculaire et Cellulaire, Strasbourg, France), were maintained on a B6 background (K/B). Arthritic mice (K/BxN) were obtained by crossing K/B and NOD (N) mice. These mice were bred and maintained under specific pathogen-free conditions at the Clinical Research Institute, Seoul National University Hospital. All *in vivo* experiments were approved by the Institutional Animal Care and Use Committee of the Clinical Research Institute, Seoul National University Hospital and conducted in accordance with relevant guidelines and regulations.

### Establishment of the primary NKT cell line and coculture with bone marrow-derived dendritic cells (BMDCs)

The NKT cell line was established as reported previously^39^. Briefly, the sorted α-GalCer/CD1d tetramer^+^ TCRβ^+^ NKT cells from liver mononuclear cells were stimulated with anti-CD3 (3 μg/mL) and anti-CD28 antibodies (1 μg/mL) for 3 days and then expanded with mouse recombinant IL-2 (10 ng/mL) and IL-7 (10 ng/mL) (Peprotech) for 7 days in complete RPMI medium (10% fetal bovine serum, 1% penicillin/ streptomycin, 1% HEPES, 1% non-essential amino acid, 1% sodium pyruvate, 0.1% β-mercaptoethanol, and 1% L-glutamine). The culture media of *i*NKT cells were changed every 2–3 days, and all of the *in vitro* experiments were performed after at least 10 days of culture. RAW264.7 cells were maintained in DMEM (10% fetal bovine serum and 1% penicillin/streptomycin). Generation of BMDCs and coculture with NKT cell line were performed as described previously^40^.

### Antibodies and reagents

The antibodies and reagents used in the experiments were as follows: anti-CD3, anti-CD28, PE-Cy7-conjugated anti-TCR-β, anti-IFN-γ, anti-IL-4, anti-IL-2, and anti-IL-10 antibodies (BD Bioscience); anti-IL-13 and anti-IL-17 antibodies (R&D Biosystems); BODIPY-PA (D-321) and ER tracker (E34250) (Thermo Fisher Scientific); STF083010 (cat # 4509) (Tocris Bioscience); and tunicamycin (T7765) and sodium phenylbutyrate (SML0309) (Sigma-Aldrich). The APC-conjugated and α-GalCer-loaded CD1d tetramers were provided by the National Institute of Health (Bethesda, MD, USA).

### Fatty acid preparation and analysis of serum fatty acids in mice

Myristic acid (C14:0, T0502), pentadecanoic acid (C15:0, P6125), palmitic acid (C16:0, P9767), margaric acid (C17:0, H3500), stearic acid (C18:0, S4751), oleic acid (C18:1, O1008), linoleic acid (C18:2, 62240), cis-11-eicosenoic acid (C20:1, E3635), and cis-11,14-eicosadienoic acid (C20:2, E3127) were purchased from Sigma-Aldrich. These LCFAs were conjugated with non-esterified fatty acid (NEFA)-free BSA (Sigma-Aldrich, a7030). Briefly, fatty acids were dissolved in sterile water using a vortex and heated to 70 °C for 10 min. Fatty acids were conjugated to BSA in serum-free RPMI containing 5% NEFA-free BSA immediately after dissolving as described previously^41^. The conjugated-fatty acids were shaken at 140 rpm at 40 °C for 1 h before adding to the cells. Serum-free RPMI containing 5% NEFA-free BSA was used as the vehicle control. To evaluate the effects of palmitic acid *in vivo*, mice were injected with palmitic acid (15 μM) conjugated to free NEFA-free BSA. The fatty acid concentration in mouse serum after palmitic acid injection was measured using the acyl-CoA synthetase-acyl-CoA oxidase method (HR series NEFA HR, Wako).

### RNA isolation, real-time PCR, and XBP1 splicing assay

Total RNA was isolated using TRIzol reagent (Life Technologies, 15596–018), and cDNA was synthesized according to the manufacturer’s instructions (Invitrogen). The following primers were used for real-time PCR: Tbx21 forward: 5′-TTCCCATTCCTGTCCTTCAC-3′, reverse: 5′-CCACATCCACAAACATCCTG-3′. GATA-3 forward: 5′-GGAAACTCCGTCAGGGCTA-3′, reverse: 5′-AGAGATCCGTGCAGCAGAG-3′; IRE1α forward: 5′-GCAACCATCCTTTTGGCAAAT-3′, reverse: 5′-AACAGTCAAGGTTGCAGGCG-3′; XBP1 forward: 5′-GACAGAGAGTCAAACTAACGTGG-3′, reverse: 5′-GTCCAGCAGGCAAGAAGGT-3′; spliced XBP1 forward: 5′-AAGAACACGCTTGGGAATGG-3′, reverse: 5′-CTGCACCTGCTGCGGAC-3′; BiP forward: 5′-TCATCGGACGCACTTGGAA-3′, reverse: 5′-CAACCACCTTGAATGGCAAGA-3′; CCTα forward: 5′-GATGAGCTAACGCACAACTTCAA-3′, reverse: 5′-GTGCTGCACGGCGTCATA-3′; Edem1 forward: 5′-AAGCCCTCTGGAACTTGCG-3′, reverse: 5′-AACCCAATGGCCTGTCTGG-3′; Dnajc3 Forward: 5′-GGCGCTGAGTGTGGAGTAAAT-3′, reverse: 5′-GCGTGAAACTGTGATAAGGCG-3′; Sec. 61a1 forward: 5′-CTATTTCCAGGGCTTCCGAGT-3′, reverse: 5′-AGGTGTTGTACTGGCCTCGGT-3′; Sec. 63 forward: 5′-ACCTCCTTCGTGGGGCTCATC-3′, reverse: 5′-AATATTTGGCTGGGGTTTTA-3′; PERK forward: 5′-GCGTCGGAGACAGTGTTTG-3′, reverse: 5′-CGTCCATCTAAAGTGCTGATGAT-3′; ATF4 forward: 5′-GAGCTTCCTGAACAGCGAAGTG-3′, reverse: 5′-TGGCCACCTCCAGATAGTCATC-3′; CHOP forward: 5′-GTCCCTAGCTTGGCTGACAGA-3′, reverse: 5′-TGGAGAGCGAGGGCTTTG-3′; GADD34 forward: 5′-GAGGGACGCCCACAACTTC-3′; reverse: 5′-TTACCAGAGACAGGGGTAGGT-3′; ATF6α forward: 5′-TTATCAGCATACAGCCTGCG-3′, reverse: 5′-CTTGGGACTTTGAGCCTCTG-3′; GAPDH forward: 5′-GGGAAGCTCACTGGCATGG-3′, reverse: 5′-CTTCTTGATGTCATCATACTTGGCAG-3′. For the XBP1 splicing assay, cDNA was synthesized from purified total RNA using a high-capacity cDNA reverse transcription kit (Applied Biosystems, cat # 4368814). To evaluate the relative splicing of XBP1, RT-PCR was performed using the following primers to detect unspliced and spliced XBP1: forward: ACACGCTTGGGAATGGACAC, reverse: CCATGGGAAGATGTTCTGGG. Primers and probes for IL-4 (Mm00445259_m1), IFN-γ (Mm01168134_m1), TGF-β (Mm01178820M1), and TNF-α (Mm00443258_m1) were synthesized by Applied Biosystems. The levels of mRNA were normalized to those of GAPDH.

### Generation of mutagenesis and transfection

To validate the specific nucleotides on *t-bet* and *gata-3* mRNAs were required for RIDD, Wild-type and mutant form of *t-bet* (G971C C969G), *gata-3* (G1604C, C1602G) were cloned into the pIRES3-puro vector (Clontech). 1 μg of cloned plasmid vectors were transfected into DN32.D3 cells, a NKT cell hybridoma using electroporation (Neon transfection system, Invitrogen) under condition of 1100 V, 30ms. We allowed cells to overexpress cloned genes for 48 h and treated anti-CD3 + CD28 antibodies and palmitic acid or vehicle. We analyzed altered gene expression after 24 h after treatment using primers designed to recognize the sequence that include mutated region. The primers used for real-time PCR are following: Tbx21 forward: 5′-CAGGAAGTTTCATTTGGGAAG -3′, reverse: 5′-GCTGGTACTTGTGGAGAG-3′. GATA-3 forward: 5′-GAGGAGGAACGCTAATGG -3′, reverse: 5′-GATGCCTTCTTTCTTCATAGTC -3′.

### Diet compositions and feeding regimens

The CD and PRD were customized for the purpose of these experiments. The PRD contained higher levels of palmitic acid, but similar calories compared with those of the CD. The PRD was generated by adding palm oil (200 g/kg) to NCD (Purina), and thus palmitic acid comprised 47% of the total fat content in this diet. The CD was generated by adding medium-chain triglyceride oil (176 g/kg), safflower oil, and linoleic (24 g/kg), but minimal palmitic acid, to the NCD. The lipid content of the CD was determined according to the diet composition guide of Harlan Teklad (TD.05237, TD05235). The diet-fed mice were housed in a controlled environment that provided free access to water and chows. Eight-week-old mice were fed these diets for 1, 4, or 6 weeks, depending on the experiment.

### Confocal microscopic examination

The *i*NKT cell line was treated with 2 μM Bodipy-conjugated palmitic acid mixed with 1 × Hank’s Balanced Salt Solution (HBSS) containing 0.1% fatty acid-free albumin for 2 min, washed with cold wash solution (1 × HBSS containing 0.2% fatty acid-free albumin), and centrifuged at 1000 rpm for 5 min using cytocentrifuge (Wescor). Images were acquired using a laser-scanning confocal microscope A1 (NIKON). Three-dimensional image acquisition was performed using a Z-stack on each 0.40 μM panel and three-dimensional reconstruction was achieved using the NIS-Elements viewer.

### Electron microscopic examination

Cell pellets were fixed with 2.5% glutaraldehyde in 0.1 M phosphate buffer, pH 7.2 for 1 h at room temperature, washed, fixed in 2% OsO4 in 0.1 M phosphate or cacodylate buffer for 1.5 h at room temperature before embedding, and embedded with only epoxy resin. Thin sections were generated using an ultramicrotome (RMC MT-XL) and collected on a copper grid. The appropriate areas for thin sectioning were cut at 65 nm and stained with saturated 6% uranyl acetate and 4% lead citrate before examination using a transmission electron microscope (JEM-1400; Japan) at 80 kV.

### Knockdown of target genes using siRNA

Cells were transiently transfected with siRNAs targeting PERK (Sigma, NM_010121), IRE1α (Sigma, NM_023913), ATF6α (Sigma, NM_001081304), and XBP1 (Sigma, NM_013842) using the Neon transfection system (Invitrogen) under transfer conditions of 1100 V and 30 ms.

### mRNA stability assay and prediction of RNA secondary structures

To analyze mRNA stability, cells were treated with actinomycin D (Sigma, 5 μg/mL) at the indicated time points and then harvested for quantitative RT-PCR. To predict RNA secondary structures, we used a RNA secondary structure prediction tool (Sfold software) for statistical analysis of nucleic acid folding and studies of regulatory RNAs.

### K/BxN serum transfer and arthritis scoring

Mice were injected intraperitoneally with 80 μL pooled K/BxN serum on days 0 and 2. Ankle thickness was measured using calipers (Manostat). Jα18 KO mice were intravenously injected with the sorted hepatic *i*NKT cells from C57BL/6 mice 1 day before administering K/BxN serum. Joint swelling was monitored and scored as reported previously^10^.

### Histological examination

Whole knee joints and paws were collected 10 days after K/BxN serum transfer, fixed in 10% formalin, decalcified, and paraffin embedded. Sections were prepared from the joint tissue blocks and stained with hematoxylin and eosin. Microscopic evaluation was performed by two independent observers who were blinded to the experimental conditions. The degree of histological inflammation was scored according to inflammatory cell infiltration, fibroblastic proliferation, synovial hyperplasia, pannus formation, intra-articular exudate, and bony erosion. A semi-quantitative three point scale was developed, with a score of 0 representing the lowest feature level and a score of 2 representing the highest feature level. The score for each parameter was assigned, and the total scores were used for comparison.

### Statistical analyses

Statistical significance was analyzed using the Prism software (version 5.0; GraphPad Software, La Jolla, CA, USA). The unpaired two-tailed t-test was performed to compare groups. A value *P* < 0.05 was considered to indicate statistical significance.

## Electronic supplementary material

Supplementary figures
