# Supplementary material for: Palmitate inhibits arthritis by inducing t-bet and gata-3 mRNA degradation in iNKT cells via IRE1α-dependent decay
Source: Sci Rep. 2017 Nov 2;7:14940. doi: 10.1038/s41598-017-14780-4 (PMC5668299; doi:10.1038/s41598-017-14780-4)
Supplement: Supplementary file 1 — Supplementary figures [file 41598_2017_14780_MOESM1_ESM.pdf]

Palmitate inhibits arthritis by inducing *t-bet* and *gata-3* mRNA degradation in invariant NKT cells via IRE1α-dependent decay

Jae Sung Ko[2], Jae Moon Koh[1], Jae-Seon So[3], Yoon Kyung Jeon[1], Hye Young Kim[4], and Doo Hyun Chung[1, 2]

[1]Department of Pathology Seoul National University College of Medicine, Seoul, Korea
[2]Laboratory of Immune Regulation in Department of Biomedical Sciences, Seoul National University College of Medicine, Seoul, Korea
[3]Department of Medical Biotechnology, Dongguk University-Gyeongju, Gyeongju, Korea
[4]Laboratory of Immune Regulation in Department of Biomedical Sciences, Seoul National University College of Medicine, Seoul, Korea

Corresponding Author:
Doo Hyun Chung, M.D., Ph.D.
Department of Pathology Seoul National University College of Medicine and Laboratory of Immune Regulation, Department of Biosciences, Seoul National University College of Medicine
103 Daehak-ro, Jongno-gu,
Seoul 110-799, Korea
Tel: 82-2-740-8915
Fax: 82-2-743-5530
E-mail: doohyun@snu.ac.kr

Supplementary figure 1.

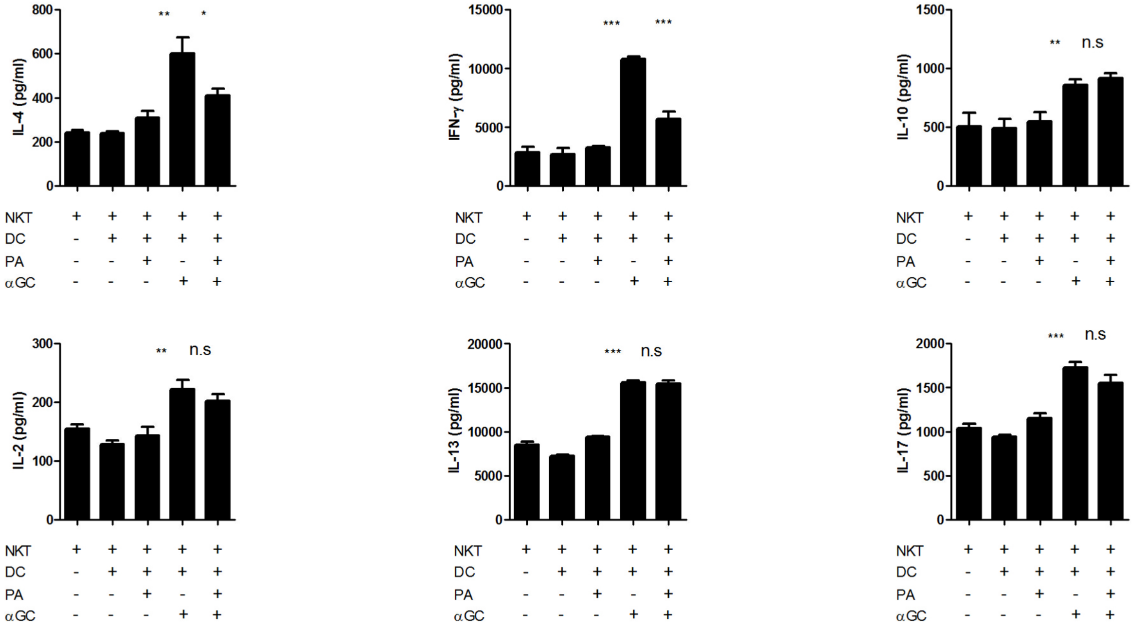

Supplementary Figure 1. Palmitic acid inhibits IL-4 and IFN-γ, but not IL-2, IL-10, IL-13, or IL-17 production by *i*NKT cells stimulated with α-GalCer. The levels of cytokines were measured in culture supernatants from an *i*NKT cell line treated with palmitic acid or vehicle in the presence of α-GalCer-loaded and irradiated bone marrow-derived dendritic cells (BMDCs) for 24 h. * $p < 0.05$, ** $p < 0.01$, ***$p < 0.005$.

Supplementary figure 2.

a

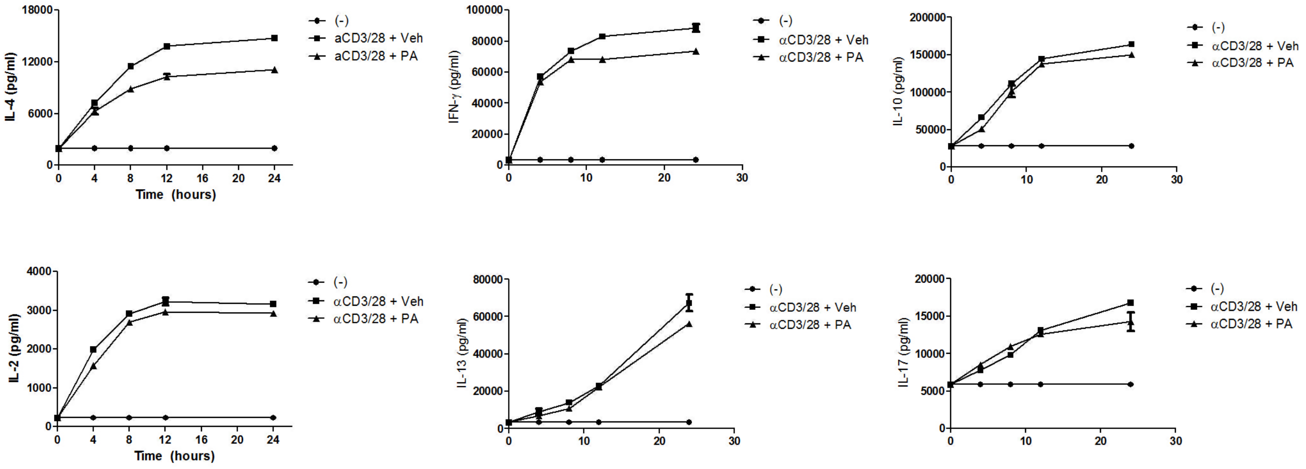

b

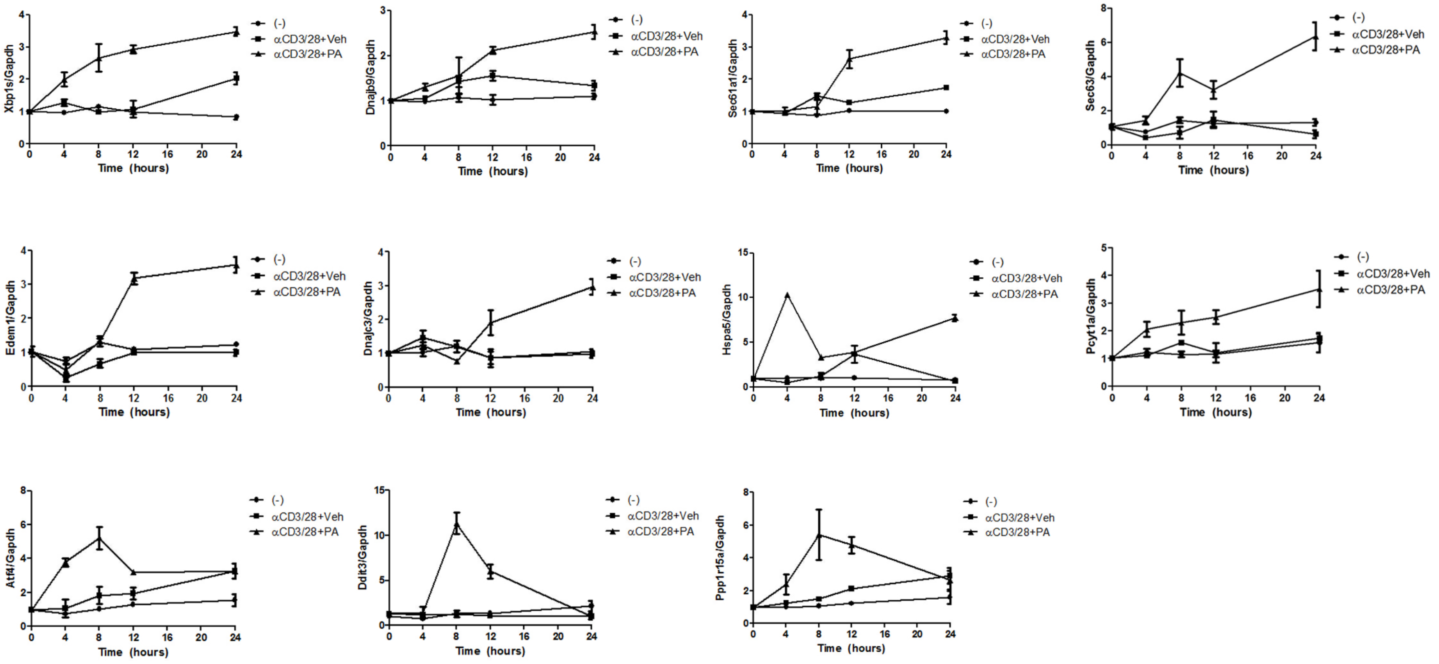

Supplementary Figure 2. The kinetic analysis for palmitic acid-mediated cytokine production and gene expression in *i*NKT cells stimulated with anti-CD3 and anti-CD28 mAb. The levels of (a) IL-4 and IFN-γ in supernatant and (b) transcript of various genes in *i*NKT cell line treated with palmitic acid or vehicle in the presence of anti-CD3 and anti-CD28 mAb were measured at various time points.

Supplementary figure 3.

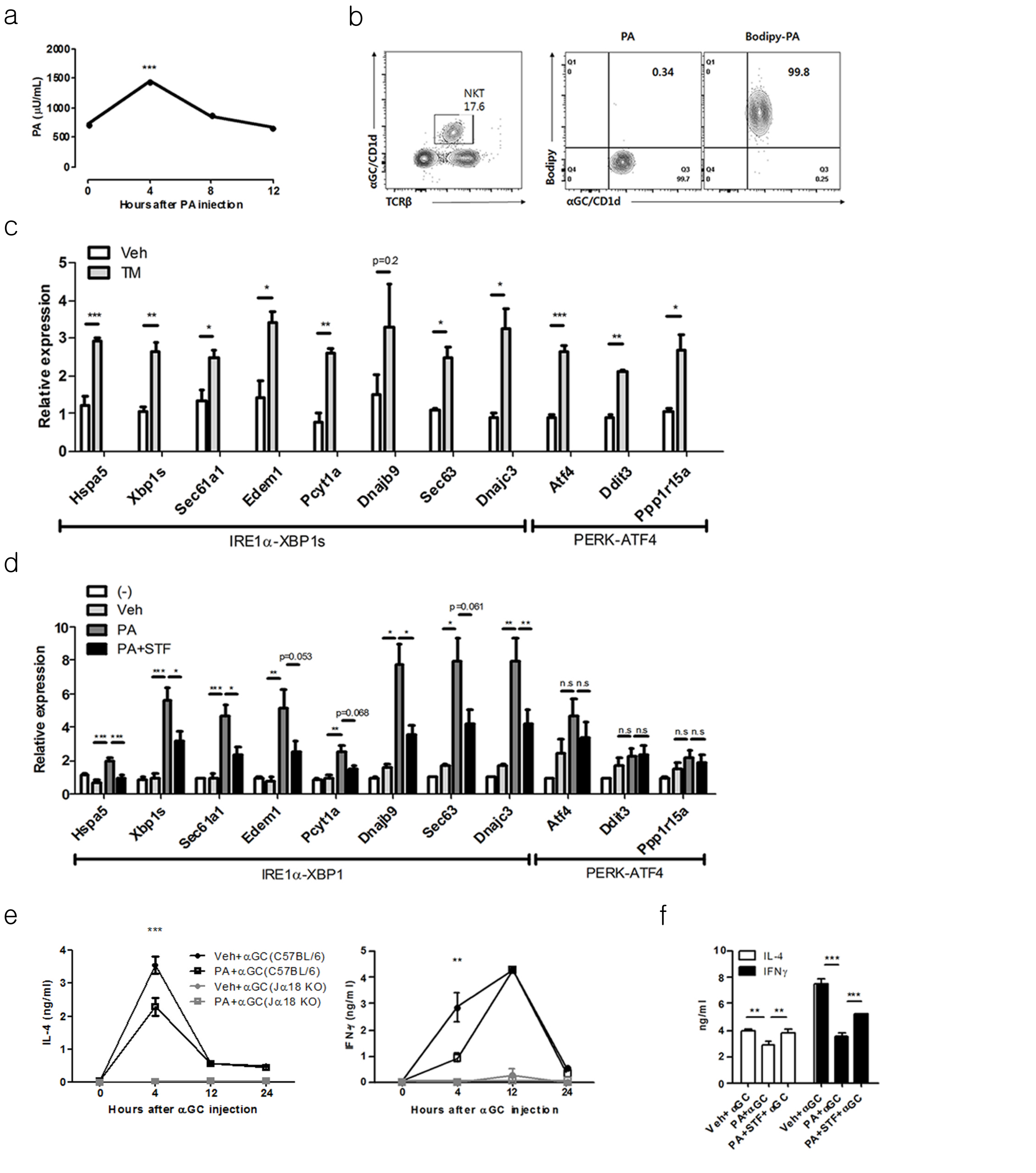

Supplementary Figure 3. Palmitic acid inhibits IL-4 and IFN-γ production by *i*NKT cells via ER stress in the presence of TCR stimulation in vivo. (a) Serum levels of palmitic acid were measured in palmitic acid-injected C57BL/6 mice at the indicated time points. (b) The levels of Bodipy-conjugated palmitic acid were estimated in hepatic *i*NKT cells obtained from C57BL/6 mice injected with Bodipy-conjugated palmitic acid using flow cytometry. (c and d) The expression levels of Hspa5, spliced xbp-1, Sec61a1, Edem1, Pcyt1a, Dnajb9, Sec63, Dnajc3, Atf4, Ddit3, and Ppp1r15a in hepatic *i*NKT cells obtained from C57BL/6 mice injected with (c) vehicle or TM, or with (d) vehicle, palmitic acid, or palmitic acid and STF083010 using real-time PCR. (e) The levels of IL-4 and IFN-γ in the serum of C57BL/6 or Jα18 knockout (KO) mice after α-GalCer + vehicle or palmitic acid injection. (f) The levels of IL-4 and IFN-γ in the serum of C57BL/6 or Jα18 KO mice after injection with α-GalCer + vehicle or palmitic aicd in the presence or absence of STF083010 administration. n = 6 per group in a, b, and e; n = 9 per group in c; n = 10 per group in d. Data were pooled from three independent experiments and analyzed. * p <0.05, ** p <0.01, ***p<0.005.

Supplementary figure 4.

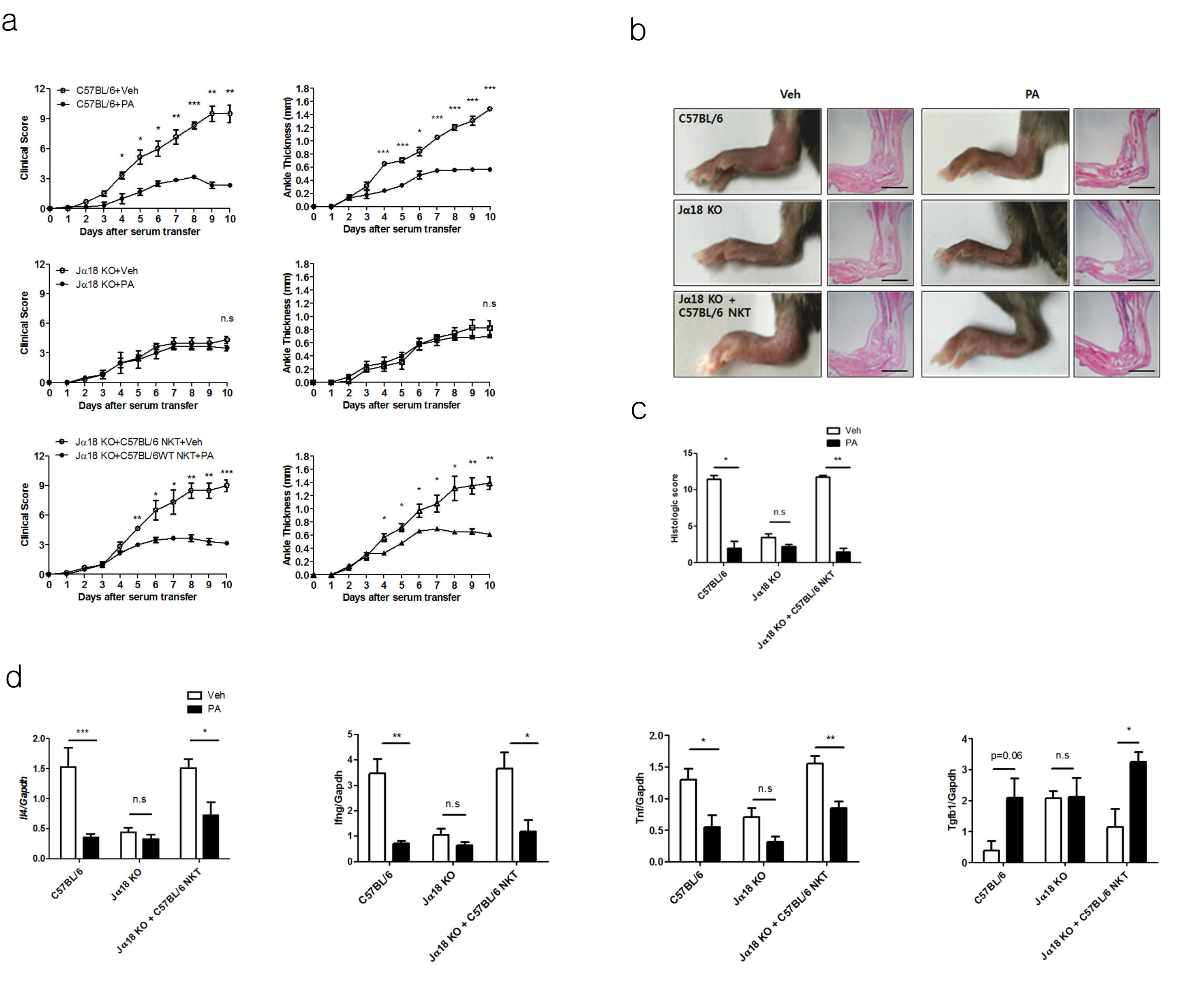

Supplementary Figure 4. Palmitic acid attenuates antibody-induced joint inflammation by inhibiting IL-4 and IFN-γ production. (a) The ankle thickness and clinical scores were measured in C57BL/6, Jα18 KO, or Jα18 KO mice with adoptive *i*NKT cell transfer. (b) The gross and microscopic images of the ankles of these mice are presented. The bars indicate 2 mm (c) The histological scores of joint inflammation were estimated. (d) The expression levels of Il4, Ifng, Tnf, and Tgfb1 were estimated in the joints of these mice during antibody-induced arthritis. n = 9 per group in a- d. Data were pooled from three independent experiments and analyzed. * p <0.05, ** p <0.01, ***p<0.005.

Supplementary figure 5.

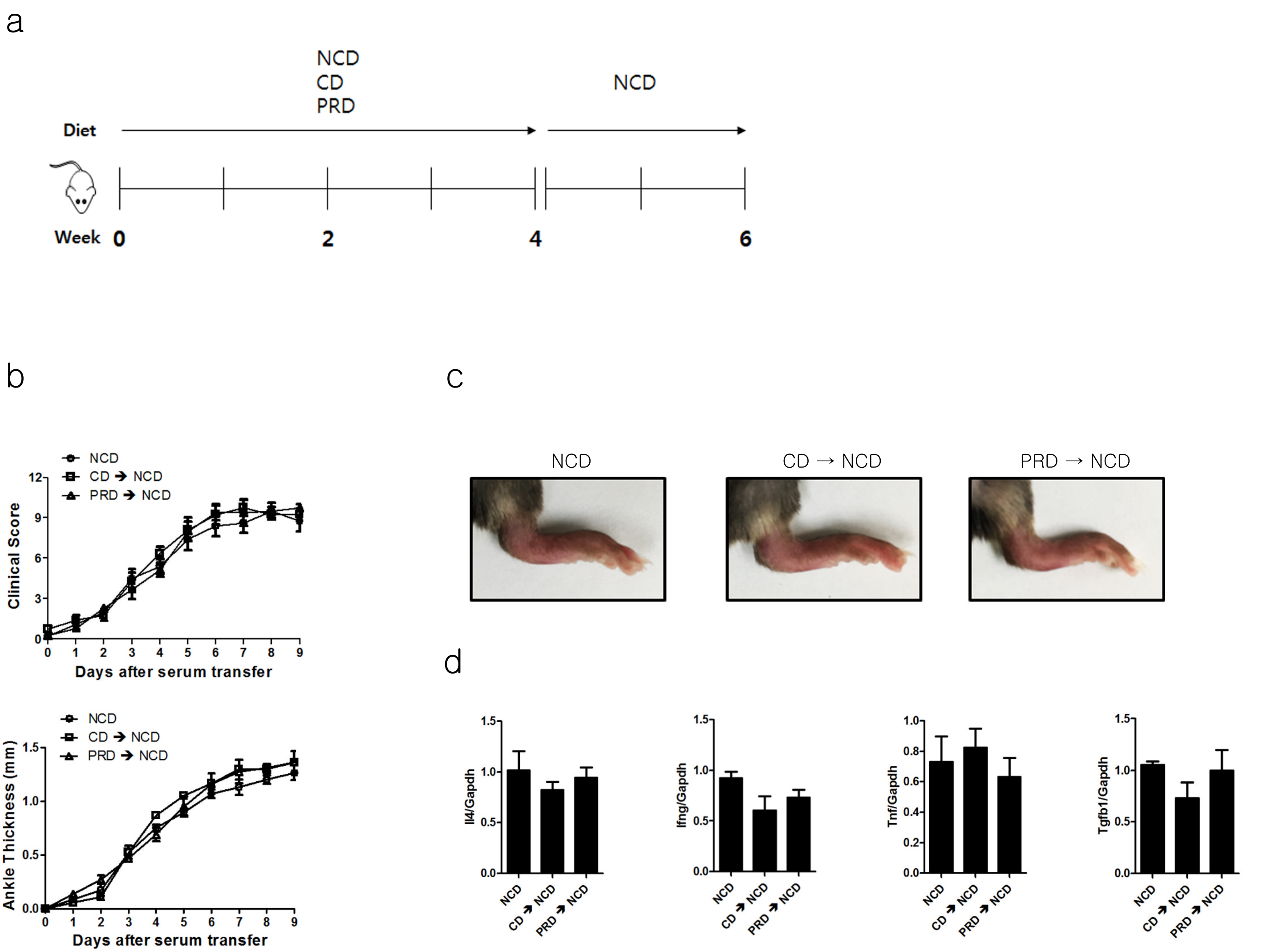

Supplementary Figure 5. C57BL/6 mice show a bouce back effect on joint inflammation once palmitic acid levels are reduced. C57BL/6 mice were fed a palmitic acid-rich diet (PRD), normal chow diet (NCD) or a control diet (CD) for 4 weeks, and then continuously fed a NCD for 2 weeks. These mice were injected with K/BxN serum to induce arthritis after 6 weeks feeding. (a) Diagram of the experimental scheme for feeding mice. (b) The ankle thickness and clinical scores were measured during antibody-induced arthritis. (c) The gross images of the ankles of these mice are presented. (d) The expression levels of Il4, Ifng, Tnf, and Tgfb1 were measured in the joints of these mice during antibody-induced arthritis. n = 10 per group in b – d. Data were pooled from two independent experiments and analyzed.
